# Retraction: MiR-211-5p Inhibits the Biological Behaviors of Colorectal Cancer via SPARC-Related Growth Factor Pathways

**DOI:** 10.7150/jca.73906

**Published:** 2022-04-26

**Authors:** Journal of Cancer

This article (MiR-211-5p Inhibits the Biological Behaviors of Colorectal Cancer via SPARC-Related Growth Factor Pathways. *J Cancer* 2022; 13(6):1895-1904. doi:10.7150/jca.60269) has been retracted due to concerns on the originality of images. Although the authors insist on the authenticity and repeatability of their data, the editors decided to withdraw this article. The authors have been contacted and they expressed apology to the readers for any negligence and mistake in this paper.

